# Antioxidant activities, functional properties, and application of a novel *Lepidium sativum* polysaccharide in the formulation of cake

**DOI:** 10.1002/fsn3.2713

**Published:** 2022-01-20

**Authors:** Sirine Ben Slima, Naourez Ktari, Aicha chouikhi, Imen Trabelsi, Amina Hzami, Mohamed Amine Taktak, Lotfi Msaddak, Riadh Ben Salah

**Affiliations:** ^1^ Laboratory of Biotechnology Microbial Enzymatic and Biomolecules (LBMEB) Center of Biotechnology of Sfax Sfax Tunisia; ^2^ Laboratory of Enzyme Engineering and Microbiology National School of Engineering of Sfax (ENIS) Sfax Tunisia; ^3^ Department of Life Sciences Faculty of Science of Gabes Gabes Tunisia; ^4^ Society of Souad Gourmandise Sfax Tunisia

**Keywords:** antioxidant activities, cake, functional properties, polysaccharides, quality

## Abstract

A novel heteropolysaccharide, named cress water soluble polysaccharide (CWSP), was purified from *Lepidium sativum* seeds. Antioxidant activities and functional properties were characterized thermally using thermal gravimetric analysis (TGA), and the differential scanning calorimeter (DSC) results of CWSP were evaluated. The total antioxidant capacity and the metal chelating activities of CWSP at 3 mg/ml were equivalent to 116.34 µg ascorbic acid and 62.57%, respectively. As for the CWSP that was used for the production of cakes, it was thermally stable, and it presented high water (WHC) and oil holding (OHC) capacities and good emulsion properties. The samples were prepared with different levels of CWSP (0.1. 0.3, and 0.5%) and analyzed during 15 days of storage at room temperature. The obtained results indicated that the addition of CWSP had a significant effect on the texture profile, leading to the increase in all parameters in terms of hardness, springiness, cohesiveness, adhesiveness, and chewiness. Moreover, the reformulation samples presented higher a* and lower L* and b* than the control sample. The sensory evaluation showed that the formulation of cake with 0.3% of CWSP was the most acceptable. Therefore, CWSP was shown to be a new alternative for improving the quality attributes, indicating potent antioxidant activities on the shelf life during the storage of bakery foods.

## Graphical abstract

CWSP were thermally stable and presented important functional properties. CWSP presented potent antioxidant activities in vivo and in vitro. CWSP supplemented in cakes improved their quality attributes

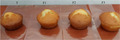

## 1 INTRODUCTION

The increasing consumer demand for food products that offer health benefits has initiated several bakery product developments, containing bioactive ingredients. It is proven that the functional product formulation by substituting or adding certain ingredients promotes health effects, such as antidiabetic, anti‐inflammatory, anticancerous, and antioxidant properties (Bhat et al., 2020).

An essential aspect in the preparation of bakery products with enhanced nutritional quality is sensory properties such as acceptability of consumer. This criterion has the priority to determine the efficient formulation of the developed products (Škrbić & Cvejanov, 2011). The presence of fat could oxidize the bakery products during storage, leading to the deterioration of the sensory properties of the final product. However, adding antioxidant compounds can prevent the autooxidation of fats and prolong the shelf life of these products. Recently, the addition of antioxidant compounds derived from plants has received much attention (Dillard & German, 2000). In this context, polysaccharides were used in food products as a source of biologically active substances due to their antioxidant and functional properties. Furthermore, these natural polymers have important bioactivities, such as anti‐inflammatories, antitumor, immunostimulator (Sun & Li, 2017; Barbosa et al., 2020) and hypocholesterolemic activities (Gil‐Ramírez et al., 2018). Polysaccharides’ bioactivities depend on various characteristics, such as monosaccharide compositions, molecular weights, and chemical structures (Ji et al., 2019).

Cress seed, *Lepidium sativum*, which belongs to the Brassicaceae family, is distributed in India, Iran, North America, and some parts of Europe. It is a seed that contains extremely nutritious substances with a pleasant aroma and flavor along with important minerals, vitamins, and metabolites, such as polysaccharides and glycoprotein complexes (Arezoo & Milad, 2018).

Polysaccharides have largely been added to food packaging (Yanming et al., 2021) and foods, as bakery, meat, and dairy products to improve their quality and provide a high sensory quality (Ben Slima et al., 2018; Xi et al., 2020). In fact, polysaccharides are used to change the textural and physicochemical properties of foods even with a very low concentration (Rongbin & Fan, 2021). Moreover, food polysaccharides usually show remarkable rheological properties, such as thickening, stabilizing, gelling, binding, and emulsifying properties of food products (Arezoo & Milad, 2018). Polysaccharides have also been successfully used to improve food shelf life via preventing settling or creaming, influencing water crystallization, retaining moisture, retarding staling, and preventing syneresis or the retro‐gradation of starch products (Xi et al., 2020). Polysaccharides extracted from byproducts can improve the cost‐effectiveness of the overall production process, and hence the final product (Ben Salah et al., 2011).

The purpose of this study was to explore the water‐soluble polysaccharide from cress seeds (CWSP) to highlight its antioxidant activities and functional properties and to evaluate its effect on cake formulations during 15 days of storage.

## 2 MATERIALS AND METHODS

### 2.1 Materials and reagents

*L*. *sativum* seeds were purchased from the market of Sfax city in Tunisia. They were crushed by a Moulinex blender LM 241, and then sieved with a 0.2 mm mesh size. The powder was kept in a well‐sealed amber glass container to protect it from sunlight. The cupcake ingredients were obtained from Gourmandise (Sfax‐Tunisia).

### 2.2 **Extraction of water‐soluble polysaccharide from** *L*. *sativum* **(CWSP)**

Water‐soluble polysaccharide from cress seeds (CWSP) was purified according to the method of Ben Slima et al. (2018). Briefly, ethanol was used to pre‐extract and remove cress seed powder pigments. The extract was obtained after 20 volumes of deionized water addition at 90°C while being stirred for 4 hr. The obtained filtrates were evaporated under vacuum. The concentrated liquid was then precipitated at 4°C for 24 hr using 95% (v/v) ethanol. The final purified polysaccharide (CWSP) was later on lyophilized and stored at 4°C.

### 2.3 Thermal analysis of CWSP

#### 2.3.1 DSC measurements

The phase transition was determined using DSC analysis. The thermal analysis of CWSP was tested with a differential scanning calorimeter from −2.77 to 250°C (Mettler Toledo 181 Star) at a rate of 5°C/min.

#### 2.3.2 TGA Thermo‐gravimetry analysis

Thermo‐gravimetric analyzer (Mettler Toledo TGA/SDTA 8951E) was used to determine the thermal gravimetric analysis (TGA). Five milligrams of the sample was introduced into the sample pan and heated from 30 to 400°C under nitrogen atmosphere. The gas flow rate was 40 ml/min.

### 2.4 Functional properties of CWSP

#### 2.4.1 Water‐holding capacity

The water‐holding capacity (WHC) was recorded by the method of Trigui et al. (2018). One gram of CWSP was dispersed in distilled water (25 ml). The suspensions were held at different temperatures (25, 50, or 75°C) for 1 hr. Besides, the excess water was drained at 50°C for 25 min. The WHC was calculated according to the following formula:

$$WHC=\frac{weight\;of\;the\;\;tube\;content\;after\;draining}{weight\;of\;the\;dried\;SWPS}$$

The WHC was expressed as g of the absorbed water per g of the sample.

#### 2.4.2 Oil‐holding capacity

The oil‐holding capacity (OHC) was determined following Trigui et al. (2018). The samples (0.5 g) were poured in volumes of 10 ml of soybean oil. Then, the suspensions were kept at different temperatures (25, 50, or 75°C) for 1 hr. The obtained supernatant was removed and the excess oil was drained. OHC was calculated as the weight of the tube contents after dividing draining by the weight of the dried CWSP. OHC was expressed as g of the absorbed oil per g of sample.

#### 2.4.3 Emulsifying activities

##### Determination of emulsifying activities

CWSP emulsifying activities were assayed as reported by Ben Slima et al. (2018). Soybean oil was added to the CWSP solution (0.3%) at a ratio of 3:2 (v/v) and stirred in the vortex for 2 min at 2400 rpm. After 1, 24, and 168 hr, emulsification indices E1, E24, and E168 were calculated as follows:

$$EI=\left(\text{he}\;/\text{ht}\right)*100$$

where he (mm) is the emulsion layer height and ht (mm) is the mixture overall height after *t* hours.

##### Effects of temperature, pH, and ionic strength on emulsion stability

Soybean oil was chosen for subsequent assays. Therefore, CWSP (0.3%) emulsion stabilizing capacity was assayed at a large range of temperature (20–100°C) for 1 hr, pH (2.0–12.0), and ionic strength (0.2–2 M NaCl).

##### Microscopic assessment of emulsions

After 24 hr of emulsion storage, a light microscope (NIKON ECLIPSE E200) with a 10 × objective lens was used to examine the emulsion samples. For sample preparation, a pin‐tip amount of the emulsion was smeared on the microscope glass slide and then quickly covered with a coverslip.

### 2.5 Antioxidant activities of CWSP

#### 2.5.1 Total antioxidant activity

The total antioxidant activity of CWSP was determined by the phosphate molybdate method (Prieto et al., 1999). Briefly, 1 ml of reagent solution (0.6 M sulfuric acid, 28 mM sodium phosphate, and 4 mM ammonium molybdate) was added to 0.1 ml of samples at various concentrations (1–3 mg/ml). After incubation at 90°C for 90 min and cooling, the mixture was measured at 695 nm. The antioxidant activity was expressed as ascorbic acid equivalents. BHT was used as a positive control.

#### 2.5.2 Ferrous chelating activity

The ferrous chelating activity of CWSP at different concentrations (1–5 mg/ml) was measured according to the method of Ben Slima et al. (2019). Briefly, distilled water and FeCl_2_ (2 mM) were mixed with the sample. After 15 min, 0.1 ml of ferrozine (5 mM) was added. Finally, the Fe^2+‐^ferrozine complex absorbance was measured at 562 nm after 10 min reaction time. EDTA was used as a reference.

### 2.6 Preparation of cakes

The cakes consisted of the following ingredients: wheat flour, sugar, egg yolk, baking powder, and butter. All ingredients were obtained from Souad Gourmandise society, Sfax‐Tunisia. Wheat flour supplemented with CWSP was prepared. After mixing the ingredients, the dough samples were placed on aluminum trays and baked at 180°C for 30 min and then allowed to cool. Then, the samples were stored at room temperature under natural relative humidity conditions in a closed bag for storage studies (0–15 days) until analysis. Each analysis was performed on three independent experiments which were evaluated in triplicate.

Four formulations were prepared: C: control formulation without polysaccharides; T1: formulation with 0.5% of CWSP; T2: formulation with 0.3% of CWSP; and T3: formulation with 0.1% of CWSP.

### 2.7 Antioxidant activities of cake

DPPH•radical‐scavenging, ABTS, and reducing power were measured in cakes as previously described (Msaddak et al., 2015). Five grams of bread sample was homogenized with 25 ml ethanol for 2 hr at 25°C using an orbital shaker at a stirring speed of 200 rpm. After centrifugation at 8000 rpm for 30 min, the supernatant was recovered. The DPPH radical‐scavenging activity (%), the ABTS radical‐scavenging activity (%), and the reducing power (absorbance at 700 nm) of cakes were measured as previously described (Msaddak et al., 2015). The determination of antioxidant activities was carried out in triplicate.

### 2.8 Thiobarbitric acid assay (TBA)

TBA was based on a method adapted from Bajaj et al. (2016). Briefly, 10 g of ground cookies samples was mixed in 25 ml of distilled water and 10% trichloroacetic acid. To 1 ml of filtrate, an amount of 3 ml of TBA solution (0.67%) and 0.05 N of H_2_SO_4_ was added and the mixture was heated on a water bath at 95℃ for 30 min. After cooling, n‐butanol (4 ml) was added and centrifuged at 1500 rpm for 10 min. The organic layer was pipetted out and the absorbance was measured at 532 nm. The determination of TBA was carried out in triplicate.

### 2.9 Texture profile analysis (TPA)

The crumb texture was evaluated for cakes stored at 25°C for 24 hr and 72 hr using a Texture Analyzer (Model TA.XT*plus,* Stable Microsystems,). The texture profile analysis (TPA) for cakes was determined by a texture analyzer (TA‐XT2i, Stable Micro Systems Ltd.,) and performed at room temperature (Moza & Gujral, 2017). Texture parameters (hardness, springiness, cohesiveness, and chewiness) were quantified during 10 days of storage for 20 mm‐thick cake slices using the TPA double compression test. Actually, the samples were compressed to 50% compression at a speed of 10 mm/sec and a 5 s delay between the first and second compression. The determination of TPA was carried out in triplicate.

### 2.10 Determination of color of cakes

The color parameters of crumb cakes (lightness L*, redness a*, and yellowness b*) were determined with a Color Flex spectrocolorimeter (Hunter Associates Laboratory Inc.,) for 10 days of storage at room temperature (Ben Slima et al., 2017). The determination of color was carried out in triplicate.

### 2.11 Sensory evaluation

The sensory properties (texture, color, odor, taste, and overall acceptability) of freshly prepared cakes were evaluated by 60 panelists. A nine‐point hedonic scale with 1 (dislike extremely), 5 (neither like nor dislike), and 9 (like extremely) was used. Water was provided to rinse the mouth between evaluations.

### 2.12 Statistical analysis

One‐way analysis of variance (ANOVA) was performed using the SPSS program (V17.0) to determine the significant differences between treatments. The results were expressed as mean values and standard errors from three replications. All tests were performed at the 0.05 level of significance.

## 3 RESULTS AND DISCUSSION

### 3.1 DSC measurement

**FIGURE 1:**
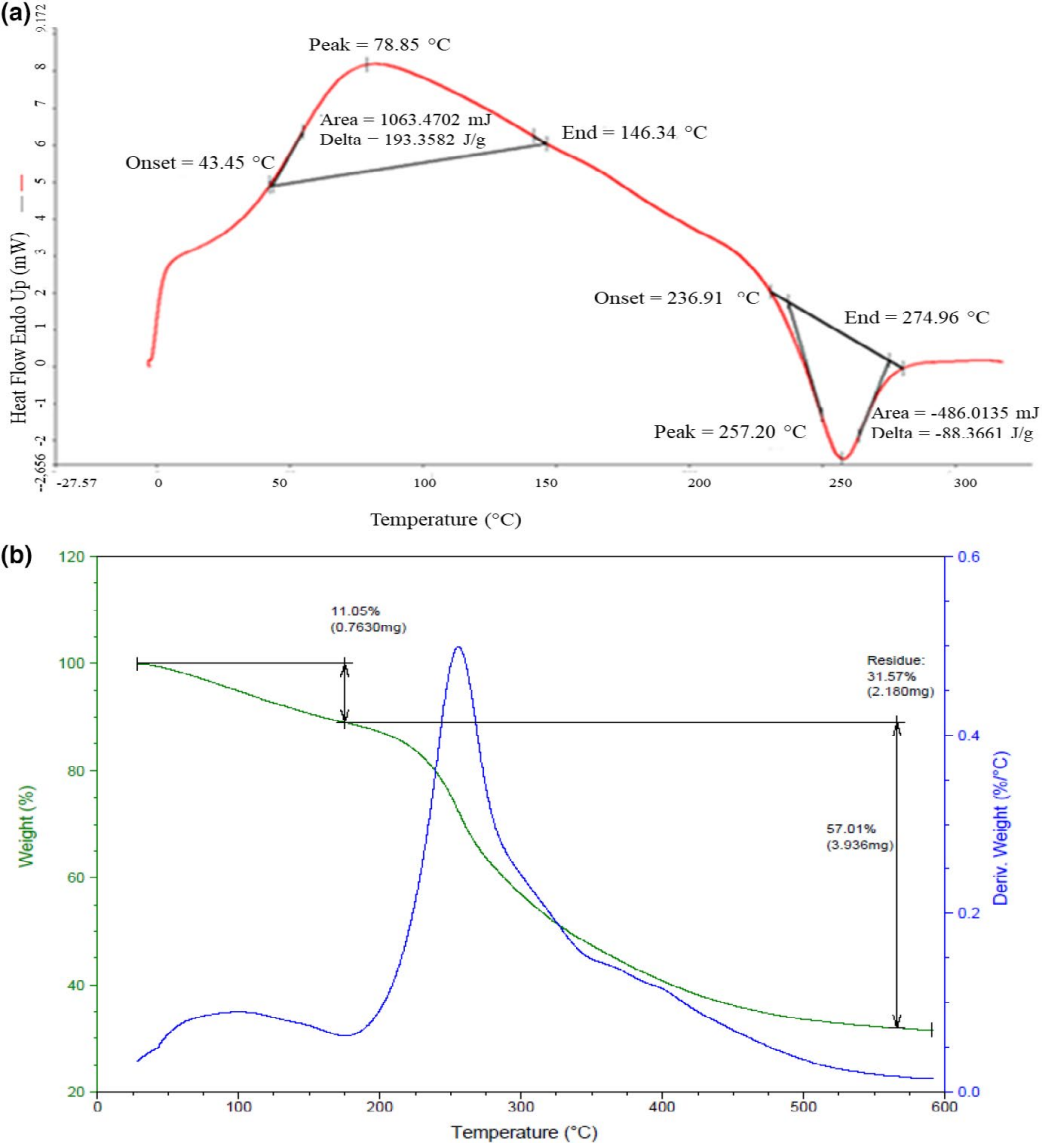
Thermal analysis: of CWSP: (a) DSC; (b) TGA

Differential scanning calorimetry (DSC) is considered a powerful technique to examine chemical and physical changes allowing to determine the kinetics of polysaccharide polarizing during thermal processing. As shown in Figure 1a, an obvious endothermic peak at 87.83°C in DSC curve was detected, indicating the dehydroxylation reaction and the loss or the dehydration of peripheral polysaccharide chains (Jalaleldeen et al., 2020). Besides, an exothermic peak was observed at 274.96°C, indicating the level of cross‐linking of polymers (Matheus, 2016). A similar curve was observed in the case of tamarind seed polysaccharide, which seems to behave completely differently; its endothermic peaks were indicated at 85.29°C and 270.78°C and it was followed by an exothermic peak at 318°C (Ktari et al., 2020). Other studies reported that polysaccharides extracted from *Sorghum bicolor* seeds, *D*. *melonoxylon, and B*. *lanzan* had transition temperatures of 78.85°C, 78°C, and 89°C, respectively (Ben Slima et al., 2018; Bothara & Singh, 2012). In addition, Ktari et al. (2020) reported that the DSC thermogram of senegrain polysaccharides presented a wide endothermic peak at 90.11°C and a small endothermic peak at 270.78°C. The different thermal transitions are linked to the polymer chemical nature and the temperature. The polysaccharide transition temperature is also related to monosaccharide composition and polymerization degree (Ktari et al., 2020).

### 3.2 Thermo‐gravimetry analysis

TGA is a simple tool to study the thermal behavior and the decomposition pattern of polymers. In fact, it can provide the mass change measurement in materials related to decomposition pattern, dehydration, and oxidation of polymers with temperature and time.

As shown in Figure 1b, the initial weight loss at the temperature range 28.33–175°C was observed, which could be attributed to the loss of bound and free water (Kittur et al., 2002). The weight loss of CWSP was shown in the temperature range 175.00–566.03°C, which might be due to the CWSP decomposition. The results suggested that CWSP was relatively stable below 566°C. Hence, the thermal stability of CWSP may be an important functional property in some food applications.

### 3.3 Functional properties of CWSP

#### 3.3.1 Water‐holding capacity of CWSP

The WHC represents the amount of water held, used to assess the stability, sensory, and texture of sample. As shown in Table 1, the WHC values of CWSP were 15.21, 15.48, and 17.98 at 25, 50, and 75°C. CWSP was found to possess a WHC level higher than those obtained for polysaccharides isolated from black cumin seeds at different temperatures (Trigui et al., 2018). It was reported that WHC could be attributed to pore size, polysaccharide source, and the capillarity of the molecule conformational structure (Shen et al., 2019).

**TABLE 1:** Water‐holding capacity, and oil‐holding capacities of CWSP (g water or oil/g wet sample)

Temperature	25°C	50°C	75°C
WHC	15.21 ± 1.2	15.48 ± 1.1	17.98 ± 0.9
OHC	1.18 ± 0.29	1.3 ± 0.28	1.11 ± 0.28

#### 3.3.2 Oil‐holding capacity of CWSP

The OHC of CWSP was investigated at 25, 50, and 75°C. As shown in Table 1, the highest OHC value was detected at 50°C, which recorded 1.31 g oil/g sample. This value was lower than that of polysaccharides from *Lilium lancifolium* Thunb (9.25 g oil/g sample) (Gao et al., 2015), and higher than durian seed gum (0.44–1.29 g oil/g sample) (Mirhosseini & Amid, 2012). Although OHC is partially linked to chemical composition, it is closely related to fiber structure porosity and the affinity of the fiber molecule to oil (Shen et al., 2019).

#### 3.3.3 Emulsifying activities of CWSP

##### Determination of emulsifying activities

CWSP emulsifying activities, including emulsion capacity (EC) and emulsion stability (ES), at 0.3% concentration using corn oil were determined. CWSP exhibited a high rate of EC, up to 77.77% after 1 hr. The results showed that EC was related to polysaccharide composition, rheological characteristics, and the residual components of the hydrophobic protein (Ben Slima et al., 2018). After 24 hr, CWSP still formed stable emulsions as shown by the emulsification indices (E168), which reached 75.58%. Our results also revealed that ES had a positive effect on its physical stability (gravitational separation, flocculation, and coalescence) (McClements, 2005).

##### Effects of pH, temperature, and ionic strength on emulsion stability

**FIGURE 2:**
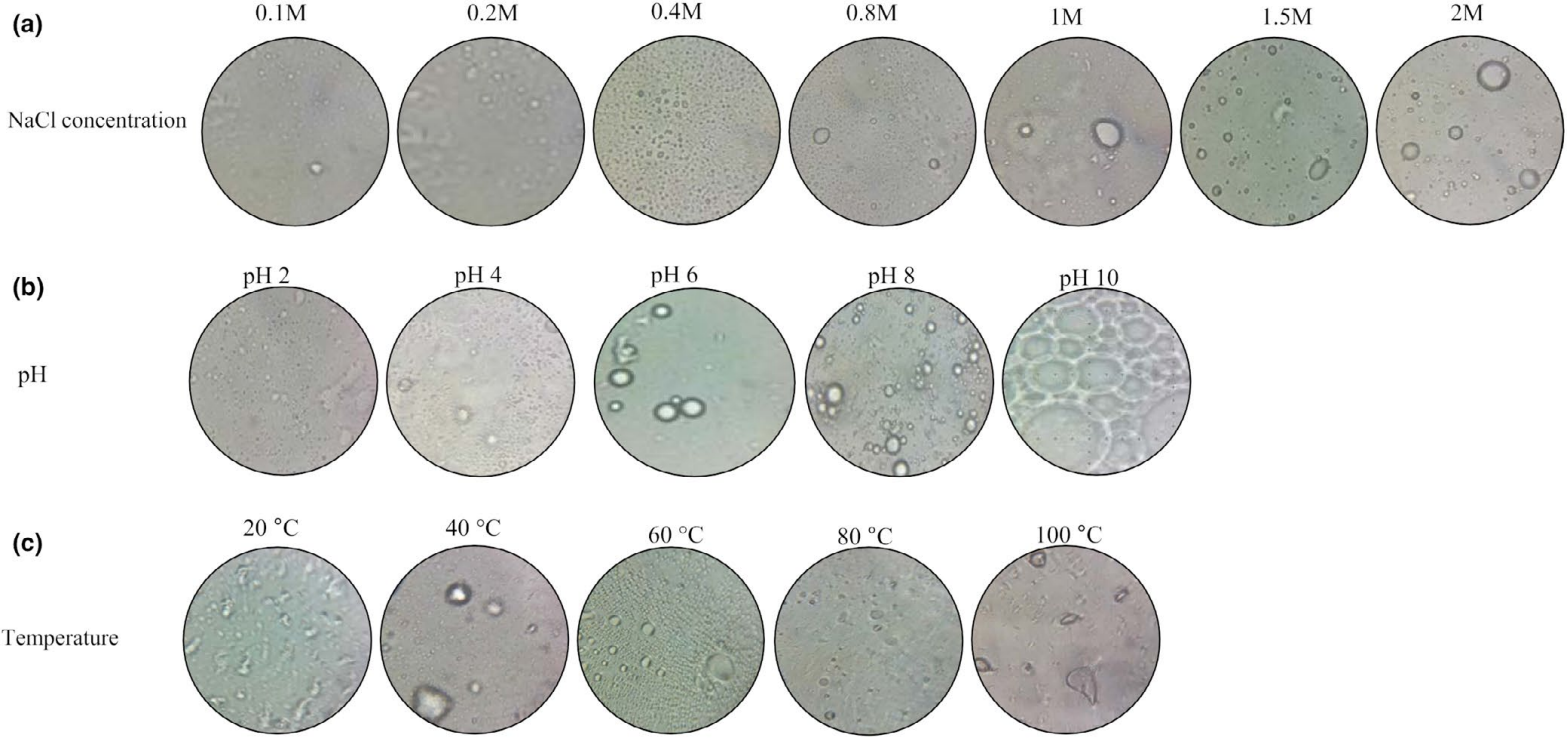
Effects of ionic strengh (a), pH (b), and temperature (c) on emulsion stability

Emulsifiers are frequently exposed to extreme ionic strength, pH, and temperature in various industrial processes. Therefore, CWSP emulsion stabilizing capacity and emulsifying activities for corn oil were tested under different conditions of NaCl concentration (0.2–2.0 M), pH (2.0–12.0), and temperatures (20–100°C) (Figure 2).

As shown by the microscopic and macroscopic appearance in Figure 2a, the emulsions of CWSP were relatively stable at low NaCl concentrations (0.1–0.4 M), which presented a monomodal size distribution, with the smallest mean droplet size of emulsions. Indeed, it is reasonable to suggest that the CWSP stability of emulsions is due to gel‐like network formation (Shao et al., 2017). Generally, Na^+^ had a larger protecting effect on the polymer chains by collapsing down the side chains (Shao et al., 2015). For emulsions prepared with corn oil, the increase in NaCl concentrations from 0.1 to 2 M caused an E24 decrease from 70.27% to 51.42%, respectively. It is worth noting that the ionic concentration up to 0.8 M and droplet coalescence were observed. This could emanate from the decrease of electrostatic repulsions between particles, due to the decrement of the original surface charge of particles (Xiao et al., 2016).

As depicted in Figure 2b, emulsions could be influenced by pH value differences altering polysaccharides’ molecular conformations. The pH effect on emulsion stabilities prepared with biopolymers is most possibly attributable to their diverse structural and chemical compositions (Ktari et al., 2017). In this study, at acidic conditions, polysaccharide molecules adsorbed onto the surfaces of the emulsion droplets were less dissociated, and therefore had a compact conformation, demonstrating its good emulsifying capacity. This is favorable for preventing emulsion droplets from coalescence (Sriprablom et al., 2019). At alkaline conditions, the emulsion prepared with polysaccharides had a more flexible conformation. Furthermore, at high pH values, the polysaccharide carboxyl groups were more dissociated, causing an increase in intramolecular repulsions, weakening the strength of the hydrated layer on the droplet surface. Thus, the coalescence of droplets became very significant (Sriprablom et al., 2019).

CWSP emulsifying properties were also evaluated against temperature changes from 20°C to 100°C (Figure 2c). After being heated to 20, 40, and 6°C, CWSP retained its ability to form stable emulsions. In fact, a decrease in the emulsification index was observed at temperatures above 60°C. Generally, CWSP stabilizing activity may be used as a relatively high temperature‐subjected emulsion stabilizer. Analyzing the data from microscopic evaluation, at 20, 40, and 60°C, emulsions presented monomodal size distribution, with the smallest mean droplet size. Consequently, it is reasonable to propose that the emulsion stability of CWSP is due to steric emulsions, which stabilize by forming an extended three‐dimensional gel‐like network in the continuous phase (Ktari et al., 2017). The larger droplet size values were detected at 80 and 100°C, which, in turn may be associated with the droplet irreversible flocculation and coalescence. Similar results were observed by Ktari et al. (2017).

### 3.4 Antioxidant activities of CWSP

#### 3.4.1 Total antioxidant activity of CWSP

**FIGURE 3:**
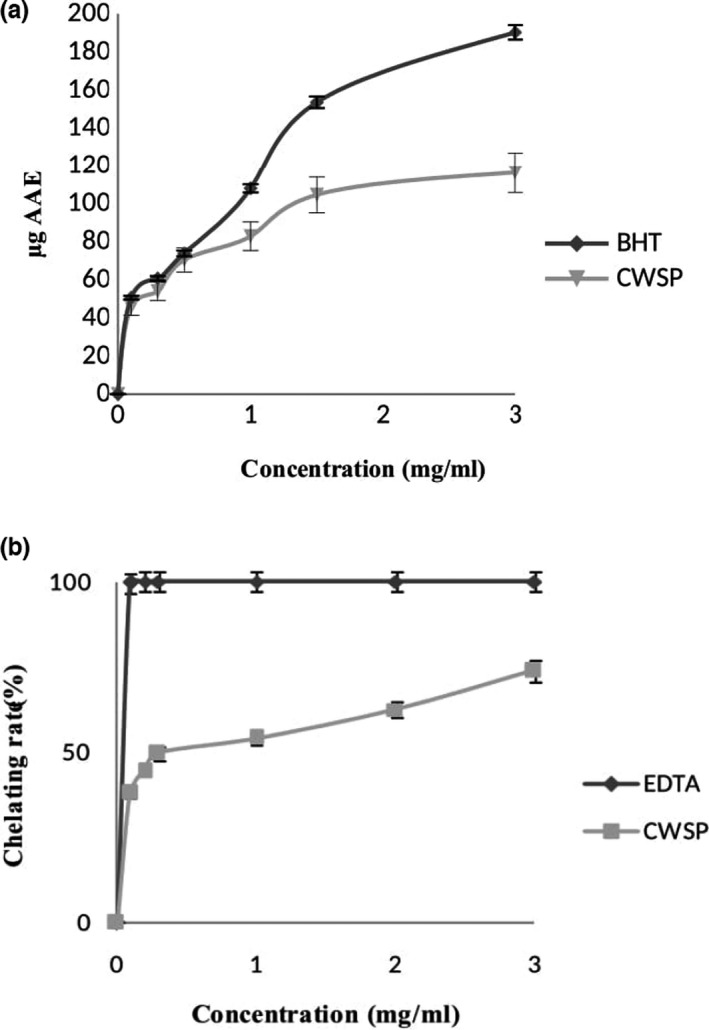
Antioxidant activities of CWSP: (a) ferrous chelating activity; (b) total antioxidant activity

The total antioxidant activity assays of CWSP at different concentrations (1–3 mg/ml) and BHT were expressed as ascorbic acid equivalent (µg AAE) (Figure 3a). These results indicated a positive dose–effect relationship between the antioxidant activity and sample concentrations.

Hence, the total antioxidant capacity reached a maximum value (116.34 µg AAE) at 3 mg/ml. Moreover, the CWSP presented higher values of antioxidant activities than polysaccharides from *Sorghum bicolor* seeds (Ben Slima et al., 2018).

#### 3.4.2 Chelating ability of CWSP

Ferrous ion is generally considered as the most potent pro‐oxidant that accelerates the reaction oxidation through Fenton reaction (Heng et al., 2019). The chelating ability of CWSP at different concentrations is presented in Figure 3b. In fact, the metal chelating activities of CWSP were kept dose dependent in the concentration ranges tested with a rate of 62.57% at 3.0 mg/ml. The obtained values were higher than those found by Ktari et al. (2017) and Ben Slima et al. (2019).

### 3.5 Antioxidant activities in cake

**FIGURE 4:**
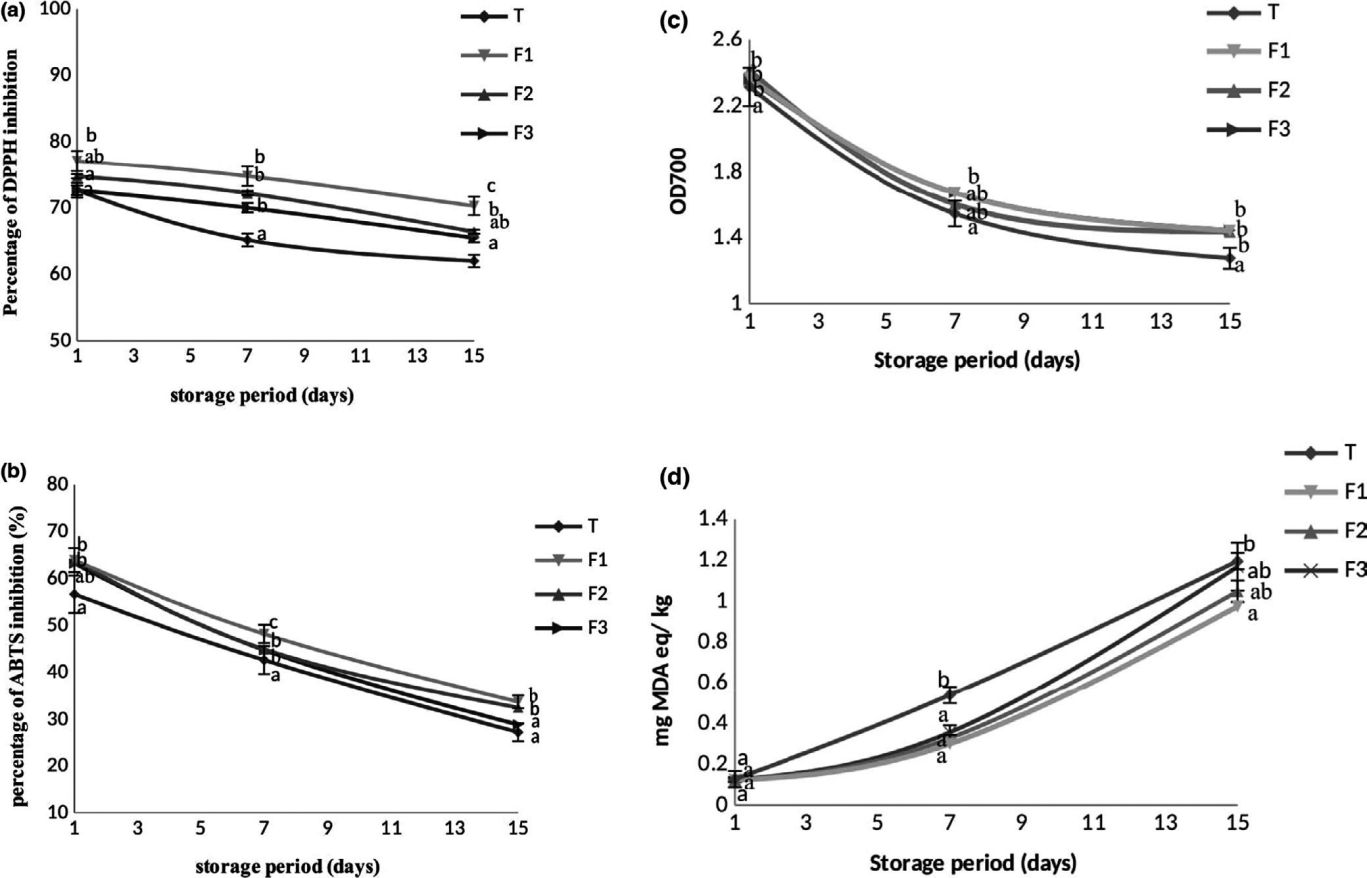
Antioxidant activities of cakes during storage period: (a) DPPH radical scavenging activity; (b) ABTS radical scavenging activity; (c) reducing power activity (OD_700nm_); (d) TBA values. Different letters indicate significant differences between samples (*p* < .05). Values are means ± *SD*

The DPPH radical scavenging activities of the cake samples are shown in Figure 4a. Cakes prepared with 0.5% of CWSP presented the highest DPPH radical scavenging activities with 77.07% on day 1 and 70.26% on day 15. Comparable trends were also noticed for ABTS (Figure 4b) and ferric‐reducing power assay (Figure 4c). In fact, the ABTS for control cake was 56.17 on day 1 and 27.79 on day 15, and increased to 63.94 on day 1 and 33.8 on day 15 for CWSP‐added cakes. Concerning ferric reducing power, the cakes formulated with CWSP exhibited the highest value of 2.36 on day 1 and 1.44 on day 15. The increase in the antioxidant activities of cakes could be ascribed to the CWSP antioxidant potential. During storage, a significant (*p* < .05) decrease in DPPH radical scavenging activity of control cake and cakes formulated with CWSP was observed. This decrease may be attributed to the compound degradation upon storage (Bhat et al., 2020). The most important reduction of antioxidant activity was observed in the control cake, which could affect the storage stability of the product. Interestingly, CWSP‐supplemented cakes preserved antioxidant activity after 10 days of storage. Consequently, these results suggest that the antioxidant potential of CWSP‐added cake reinforces their stabilization against oxidative damage. Similarly, Souza et al. (2012) have reported that the supplementation of *Gracilaria birdiae* polysaccharides in food enhances their antioxidant potential as well as their stabilization against oxidative damage.

### 3.6 Lipid peroxidation

Lipid peroxide formation is accompanied with a secondary end‐product, malondialdehyde (MDA), formation. As shown in Figure 4d, TBA value for reformulated cake samples (F1, F2, and F3) was lower than that for the control sample. Remarkably, CWSP decreased TBA values in comparison with those of the control throughout the storage. TBA value that is less than 0.6 mg/kg MDA for cake samples is said to be nonrancid, while values ranging between 0.65 and 1.44 mg/kg MDA are considered as rancid but still acceptable. As for the values that are higher than 1.5 mg/kg sample, they are considered rancid and unacceptable (Alshima 2012). On the 15th day of storage, the control sample was rancid but still acceptable. The control sample showed the highest TBA value compared to other reformulated cake samples, which indicated that the polysaccharide addition as antioxidant ingredient, can extend the shelf life of cake by retarding oxidation reactions.

### 3.7 Determination of color of cake formulated with CWSP

Since cake color has an important effect on consumer perception, it is necessary to determine the modification of the sample that affects the values of color parameters. The results presented in Table 2 reveal that cake incorporated with CWSP had a significant influence on color parameters. The values of a*, which ranged between 4.13 and 6.48, in the samples with CWSP (F1, F2, and F3) were significantly higher in comparison with those of the control sample. However, the results showed that the lightness (L*) and b* values decreased for the reformulated cake samples, which were found to be darker than the control sample, as shown by lower L*values, with different concentrations of CWSP. Indeed, the obtained results on the 15th day of storage demonstrated a reduction in L* value from 72.39 (control product) to 68.36 in cakes supplemented with a 0.5% level of CWSP. Similar results were found by Korus et al. (2015) who observed that linseed mucilage addition produced breads with darker color compared with the control bread. Moreover, CWSP addition (F1, F2, and F3) decreased b* values, which ranged between 27.23 and 30.59 at the end of storage. Owing to CWSP’s color, its addition had a significant effect on the color parameters of reformulated cakes. Furthermore, the color modifications of cakes might be attributed to the fact that polysaccharides underwent oxidation reactions and participated in caramelization reactions during baking (Alshimaa, 2012; Fernandes & Salas‐Mellado, 2017). Similar results were observed by Sulieman et al. (2019), who affirmed that reformulated cookies containing *Agaricus bisporus* polysaccharide flour were darker and presented lower b* values compared with the control sample.

**TABLE 2:** Color parameters (lightness, L*; redness, a*; yellowness, b*) of cakes during the storage period

Storage period (days)	Parameters of color	Samples			
		T	F1	F2	F3
1	a*	3.90 ± 0.29^a^	5.19 ± 0.31^b^	4.22 ± 0.35^a^	4.13 ± 0.48^a^
	b*	32.52 ± 1.31^c^	29.52 ± 0.79^a^	28.83 ± 0^a^	30.68 ± 0.0.1^b^
	L*	71.86 ± 0.41^c^	69.91 ± 0.23^b^	68.96 ± 0.29^a^	70.22 ± 0.12^b^
15	a*	3.02 ± 0.00^a^	6.48 ± 0.30^d^	5.72 ± 0.00^c^	4.91 ± 0.00^b^
	b*	29.66 ± 0.28^b^	27.23 ± 0.01^a^	30.04 ± 1.33^b^	30.59 ± 1.01^b^
	L*	72.39 ± 0.00^d^	68.36 ± 0.01^a^	69.90 ± 0.00^b^	70.28 ± 0.01^c^

Note

Statistical difference is shown by different letters (*p* < .05) between samples.

### 3.8 Texture analysis of cake formulated with CWSP

The results of texture analyses of reformulated and control cakes during the storage period at room temperature are illustrated in Table 3. They revealed that CWSP addition at different levels induced a significant effect (*p* < .05) in all texture parameters. In fact, compositional profile modifications of reformulated cake samples could alter textural profile due to unrestricted staling relating to physicochemical phenomena or insufficient structuring (Soukoulis et al., 2018).

**TABLE 3:** Texture parameters of cakes during the storage period

Storage period (days)	Parameters of texture	Samples			
		T	F1	F2	F3
1	Cohesiveness	0.15 ± 0.01^a^	0.23 ± 0.04^b^	0.21 ± 0.03^ab^	0.16 ± 0.03^a^
	Springiness (mm)	10.98 ± 0.99^a^	14.61 ± 0.28^c^	13.18 ± 0.05^bc^	12.31 ± 0.43^ab^
	Adhesiveness (*N*)	0.81 ± 0.07^a^	1.50 ± 0.05^c^	1.17 ± 0.03^b^	1.11 ± 0.06^b^
	Chewiness (Nmm)	8.95 ± 1.54^a^	10.06 ± 2.00^ab^	12.84 ± 1.15^b^	12.15 ± 1.89^b^
	Hardness(*N*)	5.58 ± 0.7^a^	7.37 ± 0.88^b^	6.68 ± 0.56^ab^	5.96 ± 0.54^a^
15	Cohesiveness	0.1 ± 0.01^a^	0.21 ± 0.02^b^	0.19 ± 0.04^ab^	0.17 ± 0.04^ab^
	Springiness (mm)	4.33 ± 0.42^a^	6.35 ± 0.29^b^	6.17 ± 0.56^ab^	5.88 ± 1.07^ab^
	Adhesiveness (*N*)	1.38 ± 0.03^a^	1.95 ± 0.06^c^	1.62 ± 0.04^b^	1.53 ± 0.08^b^
	Chewiness (Nmm)	6.17 ± 1.09^a^	12.27±2^b^	10.27 ± 1,15^ab^	10.31 ± 1,89^ab^
	Hardness(*N*)	6.85 ± 0.7^a^	10.33 ± 0.98^bc^	9.84 ± 0.55^b^	9.07 ± 0.89^b^

Note

Statistical difference is shown by different letters (*p* < .05) between samples.

The obtained results confirmed that the addition of increasing levels of CWSP caused an increase in cake hardness from 5.96 to 7.39 N. These values were higher than those of the control sample (5.58 N) on the first day of storage. Hardness also increased in reformulated cakes at the end of storage. Indeed, the improvement in carbohydrate content in cakes resulted in harder products. Moreover, the increase in the hardness of cakes with CWSP may be attributed to the decrease in water activity. Bhat et al. (2020) have attributed this increase to the formation of microglobules between polysaccharides and fats. As fat hinders the formation of the gluten network, its limited availability presumably increases the hardness of cakes. However, fermented biscuits supplemented with mushroom polysaccharide flours showed lower values of hardness due to the decrease in carbohydrate content and increase in dietary fibers and some organic acids, a result of the fermentation process (Suleiman et al. 2019). During the storage period, cake springiness increased for reformulated samples with the addition of CWSP, indicating a good possibility of adding polysaccharides for the preparation of cakes. Upon the storage of cakes, a decrease in springiness values was observed. Similar results were reported by Gomez et al. (2007) for yellow layer cakes. Cohesiveness values increased for reformulated cakes, with the highest value of 0.23 shown with the addition of 0.5% of CWSP. The lowest value was shown in the control sample (0.10) at the end of the storage period. The modification of cake cohesiveness may be related to cake moisture and cell circularity. Therefore, it can be inferred that cake samples containing a large number of air cells with smaller size could be more cohesive compared to cake samples, thus presenting a lesser dense structure (Moza & Gujral, 2017). Concerning chewiness, among all cakes prepared with different levels of CWSP, it displayed higher values compared with the control sample. Grigelmo‐Miguel et al. (1999) reported an increase in chewiness in muffins with dietetic fibers. Chewiness modifications could be related to the viscosity of added compounds (Moza & Gujral, 2017). Furthermore, CWSP incorporation increased adhesiveness. Hence, owing to the functional properties of polysaccharides, CWSP could improve the texture profile and stability of bakery products.

### 3.9 Sensory evaluations

**FIGURE 5:**
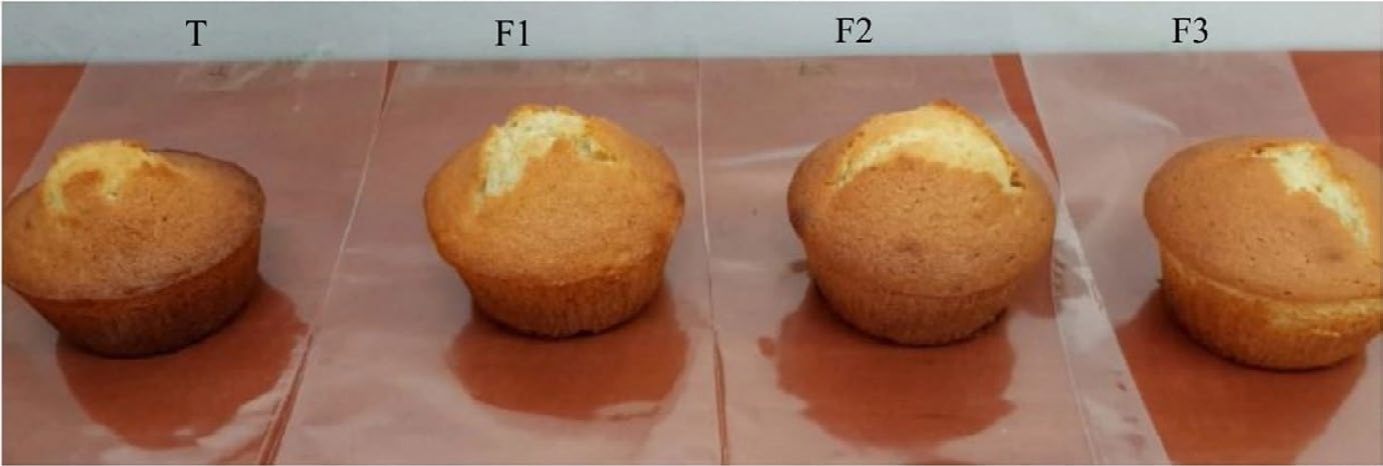
Images of the different samples of cakes formulated

The sensory evaluation of cakes was evaluated in terms of their color, texture, odor, taste, and general acceptability using the nine‐point hedonic scale (Table 4). Among the cakes, the control sample was the lowest in all sensorial attributes. Nonetheless, cake with 0.3% of CWSP (F2) was more acceptable according to panelists compared with other formulations with a better taste (Figure 5). Korus et al. (2015) demonstrated that flaxseed mucilage can be a tangible solution to improve the sensory properties and preserve the dough quality. However, Fan et al. (2007) suggested that *Auricularia auriculara* polysaccharide flour added in the baked products does not affect the sensory evaluation of the final product.

**TABLE 4:** Sensorial properties of cakes

Parameters of sensorial analysis	Formulations			
	T	F1	F2	F3
Appearance	5.90 ± 0.56^a^	6.18 ± 0.41^ab^	8.00 ± 0.69^c^	7.37 ± 0.65^bc^
Color	5.68 ± 0.25^a^	6.93 ± 0.13^b^	6.81 ± 0.22^b^	6.48 ± 1.00^b^
Texture	5.84 ± 0.23^a^	7.22 ± 0.40^b^	7.18 ± 0.78^ab^	6.62 ± 0.75^ab^
Odor	5.62 ± 1.31^a^	5.75 ± 1.57^a^	6.37 ± 1.31^a^	6.31 ± 1.40^a^
Taste	6.08 ± 0.48^a^	6.29 ± 0.76^ab^	7.28 ± 0.94^ab^	7.66 ± 0.35^b^
General acceptability	6.89 ± 0.31^a^	6.87 ± 0.99^a^	8.18 ± 0.96^c^	7.46 ± 0.53^b^

Note

Statistical difference is shown by different letters (*p* < .05) between samples.

## 4 CONCLUSION

Polysaccharides extracted from cress seeds showed potent antioxidant activities and important functional properties. The obtained results showed that CWSP was used as a readily accessible source of natural antioxidants as determined in three assay models and as food additives to improve cake quality in terms of color and texture. Sensorial analysis proved that cakes with 0.3% of CWSP represented the best acceptability by the panelists. This novel polysaccharide can be widely applied to develop bakery food with good quality.

## CONFLICT OF INTEREST

The authors declare no conflict of interest.

## AUTHOR CONTRIBUTION

**aicha choukhi:** Formal analysis (equal). **Imen Trabelsi:** Formal analysis (equal). **amina hzami:** Formal analysis (equal). **mohamed amine Taktak:** Funding acquisition (equal); Validation (equal). **lotfi msaddak:** Formal analysis (equal); Validation (equal).

## Data Availability

A data available on request of authors.
